# Effect of saffron supplementation on oxidative stress markers (MDA, TAC, TOS, GPx, SOD, and pro-oxidant/antioxidant balance): An updated systematic review and meta-analysis of randomized placebo-controlled trials

**DOI:** 10.3389/fmed.2023.1071514

**Published:** 2023-02-01

**Authors:** Ali Abedi, Hassan Ghobadi, Afshan Sharghi, Sohrab Iranpour, Mehdi Fazlzadeh, Mohammad Reza Aslani

**Affiliations:** ^1^Department of Physiology, Faculty of Medicine, Ardabil University of Medical Sciences, Ardabil, Iran; ^2^Lung Diseases Research Center, Ardabil University of Medical Sciences, Ardabil, Iran; ^3^Department of Internal Medicine, Faculty of Medicine, Ardabil University of Medical Sciences, Ardabil, Iran; ^4^Department of Community Medicine, Faculty of Medicine, Ardabil University of Medical Sciences, Ardabil, Iran; ^5^Social Determinants of Health Research Center, Ardabil University of Medical Sciences, Ardabil, Iran; ^6^Applied Biomedical Research Center, Mashhad University of Medical Sciences, Mashhad, Iran

**Keywords:** *Crocus sativus*, oxidative stress, meta-analysis, saffron, malondialdehyde, total antioxidant capacity

## Abstract

**Introduction:**

This study aimed to perform an updated systematic review and meta-analysis to evaluate the effectiveness of saffron supplementation on oxidative stress markers [malondialdehyde (MDA), total antioxidant capacity (TAC), total oxidant status (TOS), glutathione peroxidase (GPx), superoxide dismutase (SOD), and prooxidant/antioxidant balance (PAB)] in randomized controlled trials (RCTs).

**Methods:**

We searched PubMed/Medline, Web of Science, Scopus, Cochrane CENTRAL, and Google Scholar until December 2022. Trial studies investigating the effects of oral saffron supplements on MDA, TAC, TOS, GPx, SOD, and PAB concentrations were included in the study. To analyze the results, mean differences (SMD) and 95% confidence intervals (CI) were pooled using a random effects model. Heterogeneity was assessed using the Cochrane *Q* and *I*^2^ values. Sixteen cases were included in the meta-analysis (468 and 466 subjects in the saffron and control groups, respectively).

**Results:**

It was found that saffron consumption caused a significant decrease in MDA (SMD: −0.322; 95% CI: −0.53, −0.16; *I*^2^ = 32.58%) and TOS (SMD: −0.654; 95% CI: −1.08, −0.23; *I*^2^ = 68%) levels as well as a significant increase in TAC (SMD: 0.302; 95% CI: 0.13, 0.47; *I*^2^ = 10.12%) and GPx (SMD: 0.447; 95% CI: 0.10, 0.80; *I*^2^ = 35%). Subgroup analysis demonstrated a significant reduction in MDA levels in studies with a saffron dosage of >30 mg/day, age of <50 years, and study duration of <12 weeks. Among the limitations of the study, we can point out that the studies were from Iran, the different nature of the diseases included, and were not considered of some potential confounders such as smoking, physical activity, and diet in the studies.

**Discussion:**

In summary, the results showed that saffron has beneficial effects on oxidative stress markers.

## Introduction

Oxidative stress is caused by an imbalance between the production of free radicals and peroxidants as well as the antioxidant defense system (1). Under mild oxidative stress, tissues counteract the effects of oxidative stress by antioxidant defense, while under severe oxidative stress conditions, biological damage and even cell death may occur (2). The sources of reactive oxygen species (ROS) are both environmental and cellular. Environmental ROS sources include industrial pollution, smoking, exhaust fumes, and occupational exposure to dust, and cellular sources of ROS include activation of xanthine oxidase (XO), nicotine adenine disphosphonucleotide (NADPH) oxidase, superoxide dismutase-1 (SOD-1), and nitric oxide synthase (NOS) (3). On the other hand, important antioxidant factors include glutathione peroxidase (GPx), glutathione reductase (GR), and catalase (3). Increased ROS production inhibits various intracellular antioxidant mechanisms. It leads to oxidative damage to nucleic acids, DNA, proteins, and membrane lipids and disrupts cellular processes, including cellular metabolism, gene expression, and cell proliferation (4).

Oxidative stress is involved in a wide range of pathological conditions. In general, it is divided into two categories based on the role of oxidative stress in the etiology of diseases. Oxidative stress is the main pathological factor in various diseases (including atherosclerosis, radiation toxicity, and paraquat toxicity). Oxidative stress is a secondary factor in disease progression [idiopathic pulmonary fibrosis, asthma, type 2 diabetes mellitus, chronic obstructive pulmonary disease (COPD), hypertension, neurodegenerative disorders, cancer, ischemia-reperfusion injury, systemic inflammatory response syndrome, and aging] (4–8).

Many recent studies have shown the effectiveness of medicinal plants for inflammatory and oxidative stress markers (9–12). Saffron (*Crocus sativus* L.) is a Mediterranean plant with nutritional and therapeutic uses. Animal and human studies have reported the beneficial therapeutic effects of saffron and its biologically active compounds (crocin, crocetin, picrocrocin, and safranal) in a variety of disorders, such as COPD, asthma, polycystic ovary syndrome (PCOS), diabetes, cardiovascular disease, metabolic syndrome, obesity, and cancer (13–19). One of the important properties of saffron is its antioxidant and anti-inflammatory effects. Recently, trial studies have examined the effects of saffron and crocin on inflammatory and oxidative stress markers in some diseases with contradictory results (17, 20). In a systematic review and meta-analysis, Morvaridzadeh et al. (21) showed the beneficial effects of saffron on oxidative stress markers [malondialdehyde (MDA) and total antioxidant capacity (TAC)]. The present study aimed to conduct an updated systematic review and meta-analysis of randomized controlled trials (RCTs) that examined the effects of saffron on oxidative stress factors, including MDA, TAC, total oxidant status (TOS), GPx, SOD, and the pro-oxidant/antioxidant balance (PAB) in various diseases.

## Materials and methods

The present systematic review and meta-analysis were performed according to the Preferred Reporting Items for Systematic Reviews and Meta-Analyses (PRISMA) strategies (22).

### Search strategy

Electronic databases PubMed/Medline, Web of Science, Scopus, Cochrane CENTRAL, and Google Scholar from inception until December 2022 were searched to find studies evaluating the effects of saffron on serum oxidant/antioxidant levels. The mesh and non-mesh terms used in the search are presented in the Supplementary Appendix. No time restrictions were applied to the search strategy. Considering that most of the trial studies on saffron have been conducted in Iran, the original research published in languages other than English and Persian were excluded from the study. Additionally, we conducted a manual search of all relevant article reference lists to identify potentially relevant trials.

### Selection criteria

The criteria of population, intervention, comparison, and outcome (PICOS) used for the present updated meta-analysis are presented in Table 1. In addition, time restrictions were not included in the study. Considering that most of the trial studies on saffron have been conducted in Iran, the original research published in languages other than English and Persian were excluded from the study.

**TABLE 1:** The population, intervention, comparison, outcome, study design (PICOS) criteria.

Criteria	Selection criteria
Population	Adults (aged ≥18 years)
Intervention	Saffron/Crocin supplement
Comparison	Placebo or no intervention
Outcome	Clinical changes in serum concentrations of oxidative stress biomarkers including TAC, TOS, CAT, MDA, NO, GSH, PAB, SOD, GPx, and isoprostanes
Study design	Randomized controlled trials

CAT, catalase activity; GPx, glutathione peroxidase activity; GSH, glutathione; MDA, malondialdehyde; NO, nitric oxide; PAB, pro-oxidant/antioxidant balance; SOD, superoxide dismutase; TAC, total antioxidant capacity; TOS, total oxidant status.

### Synthesis methods

Since crocin is one of the active ingredients of saffron, and recent clinical trial studies have focused on its effects, the present study decided that it should include both crocin and saffron trials. However, subgroup analysis of crocin and saffron was performed to evaluate the effects of each on oxidant/antioxidant markers alone. In addition to this analysis, subgroups were also performed for age, study duration, and dosage of supplementation.

### Data extraction

The data were extracted by two researchers (AA and SI), and a chief reviewer (MA) made a final decision if there was disagreement between the two researchers. The data collected from each trial were as follows: author details, mean age of participants, sex of participants, dose of saffron, number of participants in each group, study location, length of follow-up, the year the article was published, and primary outcomes. The results are reported as the mean and standard deviation for the serum levels of TAC, TOS, MDA, GPx, SOD, and PAB in the intervention and placebo groups at the beginning and end of the study.

### Statistical analysis

Results were extracted as mean ± standard deviation (SD). To calculate the standard mean difference (SMD), converted the reported results as a confidence interval, standard error (SE), minimum and maximum values, and quadratic range (IQR) to SD. Used comprehensive Meta-Analysis software version 2 and the random-effects model to analyze the results and defined statistical significance at *p* < 0.05.

Heterogeneity was evaluated using the *Q*-test and the *I*^2^ index with a significant heterogeneity level at *p* < 0.10. Subgroup analysis and sensitivity analysis to assess the impact of each study on the pooled effect size were performed. Publication bias was analyzed using the funnel plot examination and Egger’s regression test.

## Results

### Search results

**FIGURE 1:**
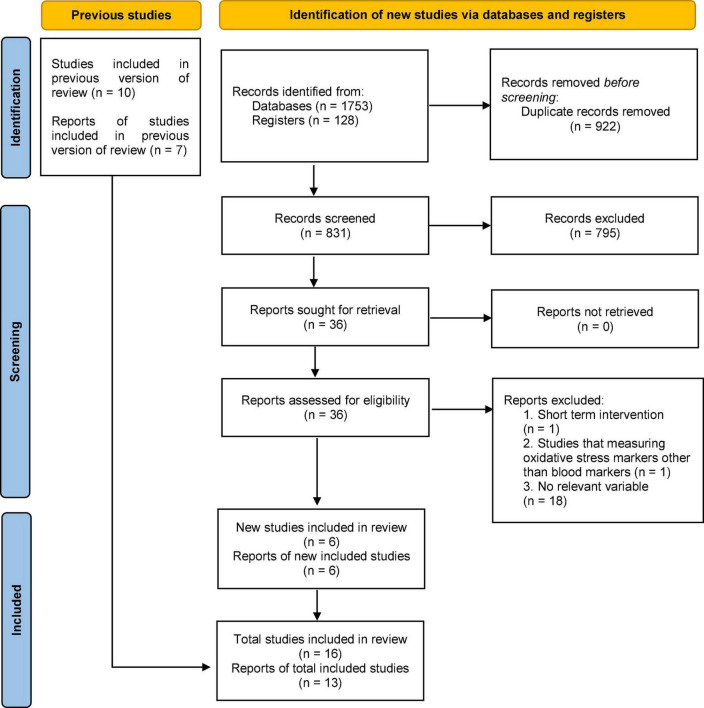
Preferred Reporting Items for Systematic Reviews and Meta-Analyses (PRISMA) flow diagram.

In the initial search, 1753 studies were extracted. We removed 922 duplicate articles and 831 articles remained for further assessment. After screening the titles and abstracts, 795 articles were removed from the study because they did not meet the inclusion criteria, such as animal studies, unrelated, and review articles. The full-text screening revealed that 18 articles could not be included in the study due to the failure to report the variables (23–40). In addition, we removed one article because the level of oxidative markers measured were saliva and urine (41). A study that had a short duration of intervention was also excluded from the study (42). It has been shown that the short duration of the intervention with saffron did not have a significant effect (43). Therefore, 16 trials were selected based on the criteria of this systematic review and meta-analysis (Figure 1). Compared to the Morvaridzadeh et al. meta-analysis, six more recent studies have been included in this meta-analysis. In the present study as well as in Marvaridzadeh, three studies were not included in the meta-analysis because they measured markers (ox-LDL, F2-isoprostanes, and DPPH) that were not reported in other studies (20, 44, 45). Finally, the current meta-analysis was performed with 13 studies (Figure 1). The selected studies were all published in English.

### Characteristics of the included studies

The characteristics of the included trials are listed in Table 2. A total of 934 subjects were recruited from the included RCTs (468 and 466 subjects in the saffron and control groups, respectively). All articles were published between 2015 and 2022, and the RCTs were performed in Iran. The duration of the intervention with saffron ranged from 4 to 12 weeks. The mean age range of the participants in the included studies was 29–55 years. Nine studies used saffron supplementation and seven used crocin supplementation for intervention. The dosage used for saffron intervention was between 30 and 1,000 mg/day, and that for crocin was between 15 and 30 mg/day. The selected trials included participants with coronary artery disease (44), type 2 diabetes (45–49), COPD (17), multiple sclerosis (50), ulcerative sclerosis (51), non-alcoholic fatty liver (52), overweight/obese prediabetic patients (20, 53, 54), rheumatoid arthritis (55), Alzheimer’s Disease (56), and methadone maintenance treatment patients (57).

**TABLE 2:** Characteristics of included studies investigating the effects of saffron supplementation on serum concentrations of oxidative stress biomarkers.

References	Country	Condition	Duration (weeks)	Group	*n*	Dose	Age, years, mean ± SD	Outcomes (change of mean ± SD)
Abedimanesh et al. (44)	Iran	Coronary artery disease	8	Saffron	22	30 mg/once daily	54.83 ± 5.99	Ox-LDL: −2.54 ± 3.30
				Placebo	20		56.00 ± 5.67	Ox-LDL: −0.24 ± 4.79
Azimi et al. (45)	Iran	Type 2 diabetes mellitus	8	Saffron	42	1,000 mg/once daily	57.02 ± 1.0	F2-isoprostan: −0.62 ± 4.00
				Placebo	39		53.64 ± 1.3	F2-isoprostan: −0.23 ± 7.15
Behrouz et al. (49)	Iran	Type 2 diabetes mellitus	12	Crocin	22	15 mg/twice daily	57.08 ± 7.41	MDA: 1.48 ± 4.98
				Placebo	22	–	59.86 ± 9.46	MDA: 1.86 ± 4.45
Dastkhosh et al. (48)	Iran	Type 2 diabetes mellitus	12	Crocin	22	15 mg/twice daily	57.08 ± 7.41	TOS: −0.82 ± 1.43
								TAC: 0.24 ± 0.39
				Placebo	22	–	59.86 ± 9.46	TOS: 0.55 ± 1.09
								TAC: −0.05 ± 0.40
Ebrahimi et al. (46)	Iran	Type 2 diabetes mellitus	12	Saffron	40	100 mg/twice daily	55.2 ± 7.3	MDA: −1.10 ± 3.41
								TAC: −0.06 ± 0.65
				Placebo	40	–	53 ± 10.6	MDA: 0.1 ± 2.7
								TAC: −0.01 ± 0.6
Ghaderi et al. (57)	Iran	Methadone maintenance treatment	8	Crocin	26	15 mg/twice daily	44.5 ± 9.4	MDA: −0.4 ± 1
								TAC: 52.30 ± 97.14
								GSH: 73.2 ± 119
								Total nitrite: 0.3 ± 5.05
				Placebo	27	–	45.6 ± 9.9	MDA: 0.1 ± 1.25
								TAC: −14.6 ± 141.75
								GSH: 21.4 ± 293.38
								Total nitrite: 1.4 ± 5.12
Ghiasian et al. (50)	Iran	Multiple sclerosis	4	Crocin	20	15 mg/twice daily	29 ± 4.99	MDA: −46.32 ± 16.75
								TAC: 24.42 ± 40.68
								TTG: 20.83 ± 15.27
				Placebo	20	–	31.47 ± 5.31	MDA: −17.94 ± 25.73
								TAC: 9.44 ± 42.69
								TTG: 4.37 ± 16.88
Ghobadi et al. (17)	Iran	COPD	12	Crocin	23	30 mg/once daily	62.04 ± 8.83	TOS: −0.14 ± 1.01
								TAC: 0.27 ± 0.51
				Placebo	23	–	61.72 ± 8.54	TOS: 0.30 ± 1.81
								TAC: −0.05 ± 0.61
Hamidi et al. (55)	Iran	Rheumatoid arthritis	12	Saffron	33	100 mg/once daily	51.55 ± 8.26	MDA: −0.54 ± 9.37
								TAC: 0.23 ± 0.94
				Placebo	32	–	51.80 ± 9.62	MDA: 2.57 ± 7.71
								TAC: 0.04 ± 1.10
Karimi-Nazari et al. (20)	Iran	Prediabetes obese individuals	8	Saffron	26	15 mg/once daily	57.95 ± 8.12	DPPH: 2.40 ± 2.02
				Placebo	30	–	57.90 ± 8.70	DPPH: −0.85 ± 2.11
Kavianipour et al. (52)	Iran	Non-alcoholic fatty liver disease	12	Saffron	38	100 mg/once daily	43.42 ± 10.62	MDA: −1.9 ± 3.51
								TAC: 0.43 ± 0.70
				Placebo	38	–	42.05 ± 8.27	MDA: −1.09 ± 2.71
								TAC: 0.08 ± 0.60
Kermani et al. (54)	Iran	Metabolic syndrome	12	Saffron	26	50 mg/twice daily	42.19 ± 11.52	PAB: −13.36 ± 26.71
				Placebo	30	–	43.60 ± 9.05	PAB: −4.19 ± 28.71
Rasi Marzabadi et al. (56)	Iran	Alzheimer disease	12	Saffron	27	15 mg/twice daily	76.70 ± 6.10	TAC: 0.18 ± 0.43
								MD: −0.15 ± 0.44
								GPx: 12.01 ± 14.97
								SOD: 124.4 ± 207.84
				Placebo	27	–	75.33 ± 5.06	TAC: 0.11 ± 0.53
								MD: −0.11 ± 0.37
								GPx: 8.99 ± 16.71
								SOD: 142.52 ± 207.11
Nikbakht-Jam et al. (53)	Iran	Metabolic syndrome	8	Crocin	29	15 mg/twice daily	38.97 ± 13.33	PAB: −16.12 ± 48.75
				Placebo	29	–	43.46 ± 12.77	PAB: −0.88 ± 36.53
Shahbazian et al. (47)	Iran	Type 2 diabetes mellitus	12	Saffron	32	15 mg/twice daily	53.5 ± 9.9	MDA: −8.94 ± 66.42
								TAC: 0.11 ± 0.43
								H-Cys: −0.12 ± 5.48
				Placebo	32	–	52.4 ± 13	MDA: −4.37 ± 63.78
								TAC: 0.1 ± 0.51
								H-Cys: −4.11 ± 6.14
Tahvilian et al. (51)	Iran	Ulcerative colitis	8	Saffron	40	100 mg/once daily	40.55 ± 12.71	MDA: −4.07 ± 12.03
								TAC: 0.11 0.88
								SOD: 5.61 ± 9.68
								GPx: 7.61 ± 15.23
				Placebo	35	–	40.97 ± 11.34	MDA: −0.11 ± 11.82
								TAC: −0.1 ± 0.90
								SOD: 0.65 ± 6.52
								GPx: −1.63 ± 10.40

DPPH, 2,2-Diphenyl-1-picrylhydrazyl; GPx, glutathione peroxidase; GSH, total glutathione; H-cys, homocysteine; MDA, malondialdehyde; Ox-LDL, oxidized LDL; PAB, proxidant/antioxidant balance; SD, standard deviation; SOD, superoxide dismutase; TAC, total antioxidant capacity; TTG, total thiol group.

### Quality assessment

**FIGURE 2:**
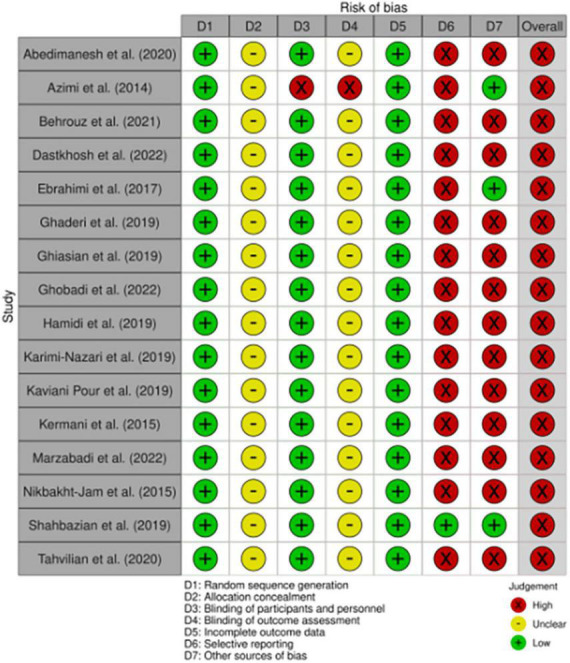
Study quality and risk of bias assessment of included studies according to the Cochrane Collaboration’s tool.

The risk of bias in eligible studies was evaluated using the Cochrane’s risk-of-bias tool for randomized trials (58). Figure 2 shows the criteria evaluated by the two researchers independently (AA and SI) for each paper included in the study.

### Qualitative results

Several oxidative stress markers were investigated in the trials but were not included in the meta-analysis due to the small number of studies. Abedimenesh et al. (44) investigated the effect of saffron supplementation on the serum levels of oxidized low-density cholesterol (ox-LDL), an indicator of oxidative stress. Their results revealed that 8 weeks of intervention with saffron (30 mg/day) significantly reduced ox-LDL levels in patients with coronary artery disease.

Another study evaluated the effect of saffron supplementation on F2-isoprostanes levels and reported that receiving saffron had no significant impact on the F2-isoprostanes concentration (45). F2-isoprostanes are considered one of the oxidative stress markers derived from arachidonic acid, the primary substance involved in lipid peroxidation. Karimi et al. (20) showed the effect of saffron supplementation on increasing the activity of diphenyl pycryl hydrazyl (DPPH) radical scavenging. Saffron exerts antioxidant effects by donating a hydrogen atom to the DPPH radical anion.

In methadone maintenance treatment patients that 8 weeks of intervention with crocin led to an increase in GSH levels (antioxidant marker) and a decrease in total nitrite (oxidant marker) levels (57). In addition, in patients with multiple sclerosis, it has been shown that treatment with crocin for 4 weeks significantly increased total thiol group (TTG) levels (50).

### Effect of saffron on MDA levels

**FIGURE 3:**
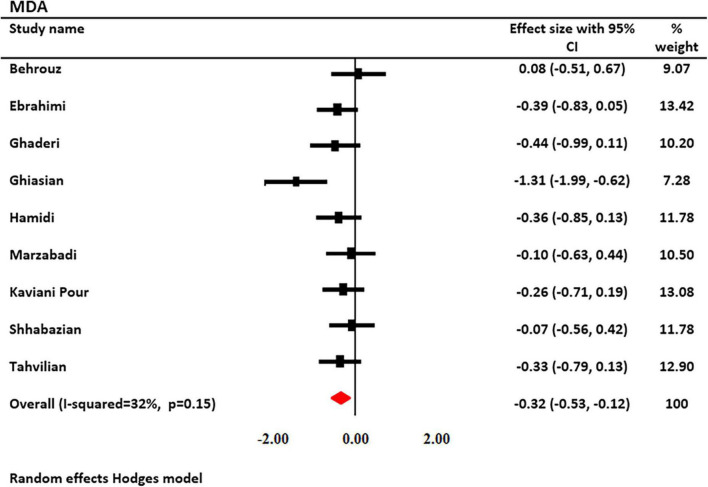
Forest plot showing the summary effect size for malondialdehyde (MDA) levels between saffron and placebo groups.

The effects of saffron and crocin supplementation on MDA levels were investigated in 9 studies (6 and 3, respectively). MDA levels were measured in 551 patients (278 patients and 273 controls). Using a random-effects model, a significant decrease in MDA levels was observed after treatment with saffron (SMD: −0.322; 95% CI: −0.53, −0.16; *I*^2^ = 32.58%, Figure 3). Decreased heterogeneity occurred when subgroup analysis was performed for study duration (*I*^2^ = 0.0%, *p* = 0.69), age (*I*^2^ = 0.0%, *p* = 0.66), type of supplementation (*I*^2^ = 0.0%, *p* = 0.98), and saffron dosage (*I*^2^ = 0.0%, *p* = 0.98).

Subgroup analysis revealed that serum MDA levels were significantly reduced following saffron supplementation in trials participants involving the following: the dosage of supplementation >30 mg/day (SMD: −0.34 mg/L; 95% CI: −0.56 to −0.11; *p* = 0.004), age of <50 years old (SMD: −0.53 mg/L; 95% CI: −0.93 to −0.13; *p* = 0.010), study duration <12 weeks (SMD: −0.50 mg/L; 95% CI: −0.95 to −0.05; *p* = 0.028), non-diabetic patients (SMD: −0.415 mg/L; 95% CI: −0.69 to 0.14; *p* = 0.003), and trials that used saffron (SMD: −0.26 mg/L; 95% CI: −0.46 to −0.07; *p* = 0.008) (Table 3).

**TABLE 3:** Subgroup analysis assessing the effect of saffron intake on MDA and TAC.

Variable	Sub-grouped by		No. of arms	Effect size (SMD)	95% CI	*I*^2^ (%)	*p* for heterogeneity
MDA	Duration	≥12ωεεκσ	5	-0.228	−0.45, −0.01	0	0.68
		<12ωεεκσ	4	-0.5	−0.95, −0.05	62.6	0.045
	Saffron dosage	>30μγ/δαψ	4	-0.335	−0.56, −0.11	0	0.981
		≤30μγ/δαψ	5	-0.335	−0.77, 0.09	65.5	0.02
	Age	<50	4	-0.526	−0.93, −0.13	57	0.073
		50≤	5	-0.196	−0.42, 0.03	0	0.66
	Supplementation type	Saffron	6	-0.262	−0.46, −0.07	0	0.908
		Crocin	3	-0.537	−1.28, 0.21	78	0.011
	Disease type	Diabetic	3	-0.169	−0.46, 0.19	0	0.408
		Non-diabetic	6	-0.415	−0.69, −0.14	41.5	0.128
TAC	Duration	≥12ωεεκσ	6	0.292	0.03, 0.55	37.1	0.159
		<12ωεεκσ	4	0.308	0.04, 0.57	0	0.746
	Saffron dosage	>30μγ/δαψ	4	0.214	−0.04, 0.47	16.9	0.306
		≤30μγ/δαψ	6	0.362	0.13, 0.60	0	0.443
	Age	<50	4	0.415	0.16, 0.67	0	0.774
		50≤	6	0.213	−0.03, 0.45	22.6	0.263
	Supplementation type	Saffron	6	0.174	−0.02, 0.37	0	0.534
		Crocin	4	0.552	0.26, 0.85	0	0.875
	Disease type	Diabetic	3	0.183	−0.27, 0.64	58	0.092
		Non-diabetic	6	0.358	0.16, 0.55	0	0.825

### Effect of saffron on TAC levels

**FIGURE 4:**
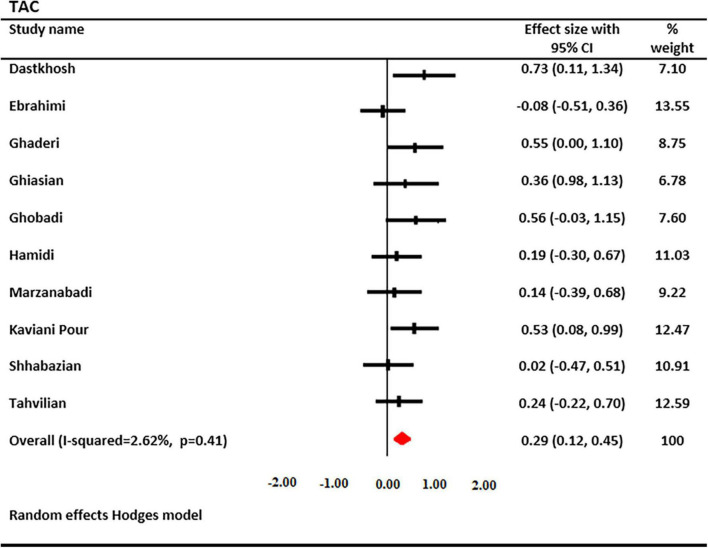
Forest plot showing the summary effect size for total antioxidant capacity (TAC) levels between saffron and placebo groups.

The effects of saffron and crocin supplementation on TAC levels were investigated in 10 studies (6 and 4, respectively). A total of 597 patients had TAC levels (301 cases and 296 controls). Using a random-effects model, a significant increase in serum TAC levels was found after treatment with saffron (SMD: 0.288; 95% CI: 0.12, 0.45; *I*^2^ = 2.62%, Figure 4). Decreased heterogeneity occurred when subgroup analysis was performed for the study duration (*I*^2^ = 0.0%, *p* = 0.74), age (*I*^2^ = 0.0%, *p* = 0.77), type of supplementation (*I*^2^ = 0.0%, *p* = 0.53), and saffron dosage (*I*^2^ = 0.0%, *p* = 0.44).

Subgroup analysis revealed that serum TAC levels were significantly increased following saffron supplementation in trials participants involving the following: the dosage of supplementation <30 mg/day (SMD: 0.36 mg/L; 95% CI: 0.13 to 0.60; *p* = 0.002), age of <50 years old (SMD: 0.42 mg/L; 95% CI: 0.16 to 0.67; *p* = 0.001), non-diabetic patients (SMD: 0.358 mg/L; 95% CI: 0.16 to 0.55; *p* = 0.000), and trials that used crocin (SMD: 0.55 mg/L; 95% CI: 0.26 to 0.85; *p* = 0.000) (Table 3).

### Effect of saffron on TOS serum levels

**FIGURE 5:**
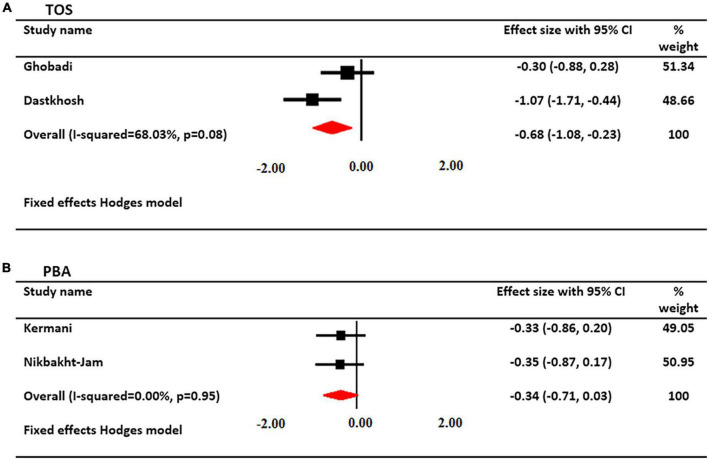
Forest plot showing the summary effect size for **(A)** total oxidant status (TOS) and **(B)** pro-oxidant/antioxidant balance (PAB) levels between saffron and placebo groups.

The effects of crocin supplementation on serum TOS levels were investigated in 2 studies. A total of 90 patients had serum TOS levels (45 cases and 45 controls). Using a fixed-effects model, a significant decrease in serum TOS levels was found after treatment with crocin (SMD: −0.654; 95% CI: −1.08, −0.23; *I*^2^ = 68%, Figure 5).

### Effect of saffron on PAB

The effects of saffron supplementation on PAB levels were investigated in 2 studies. A total of 106 patients had PAB levels (55 cases and 51 controls). Using a fixed-effects model, there was not a significant change in PAB levels after treatment with saffron (SMD: −0.342; 95% CI: −0.71, 0.03; *I*^2^ = 00%, Figure 5).

### Effect of saffron on GPx serum levels

**FIGURE 6:**
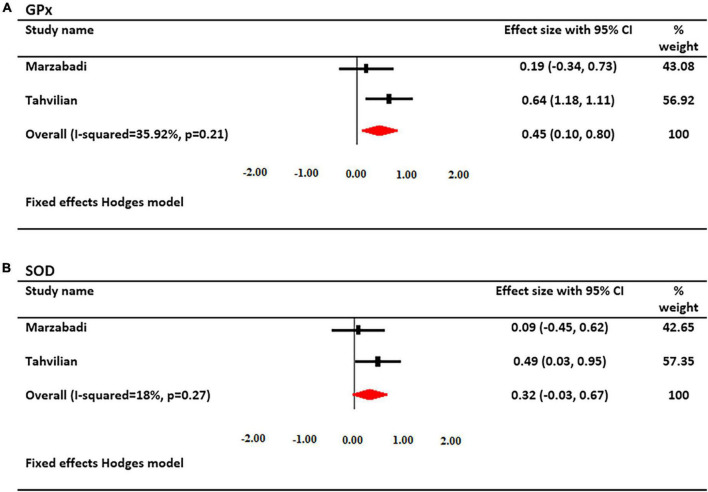
Forest plot showing the summary effect size for **(A)** glutathione peroxidase (GPx) and **(B)** superoxide dismutase (SOD) levels between saffron and placebo groups.

The effects of saffron supplementation on serum GPx levels were investigated in 2 studies. A total of 129 patients had serum GPx levels (67 cases and 62 controls). Using a fixed-effects model, a significant increase in serum GPx levels was found after treatment with saffron (SMD: 0.447; 95% CI: 0.10, 0.80; *I*^2^ = 35%, Figure 6).

### Effect of saffron on SOD serum levels

The effects of saffron supplementation on serum SOD levels were investigated in 2 studies. A total of 129 patients had serum SOD levels (67 cases and 62 controls). Using a fixed-effects model, there was not a significant change in SOD levels after treatment with saffron (SMD: 0.316; 95% CI: −0.03, 0.67; *I*^2^ = 18%, Figure 6).

### Publication bias

Visual inspection of the funnel plots did not reveal any evidence of asymmetry. Formal assessments of publication bias using Egger’s regression tests also demonstrated a lack of publication bias for both MDA (*p* = 0.23) and TAC (*p* = 0.07).

### Meta-regression

Since MDA and TAC levels were significant, meta-regression analysis was performed to determine the relationship between saffron supplement dose, saffron duration of intervention, and the age of subjects included in the study with oxidant/antioxidant markers.

Meta-regression analysis showed that there was no significant direct relationship between saffron dosage with MDA (*z* = −0.37, *p* = 0.71) and TAC (*z* = −1.58, *p* = 0.11) as well as the age with MDA (*z* = 0.88, *p* = 0.40) and TAC (*z* = −1.73, *p* = 0.08). Although there was no significant relationship between the duration of intervention with saffron and TAC (*z* = −0.52, *p* = 0.60), there was a significant direct relationship with MDA (*z* = 2.66, *p* = 0.007) (Supplementary Figure 1).

## Discussion

This study is an updated systematic review and meta-analysis of the effects of saffron on oxidative stress markers (MDA, TAC, TOS, GPx, SOD, and PAB). A combination of 16 eligible trials showed that saffron supplementation significantly reduced MDA and TOS levels, as well as increased TAC and GPx levels. In addition, subgroup analysis revealed that in trials in which the duration of administration was <12 weeks, the supplement dosage was <30 mg/day, the age of participants was under 50 years old, and used the crocin supplement, reduced MDA and increased TAC were significant events.

Saffron has long been used not only as a food source but also as a medicinal plant. In various human and animal studies, the effects of saffron are anti-inflammatory, antioxidant, anticancer, antidepressant, anti-genotoxic, analgesic, antibacterial, and respiratory relaxant (13, 23, 59). The effects mentioned above of saffron have been observed in various chronic inflammatory diseases such as asthma, COPD, cardiovascular disease, sexual dysfunction, cancer, and diabetes mellitus (14, 17, 60–62). The active ingredients of saffron with high biological activity are crocin, safranal, crocetin, and picrocrocin.

In several *in vivo* and *in vitro* studies, the anti-inflammatory effects of saffron have been attributed to its potent antioxidant and radical scavenging properties (59). Oxidative stress is caused by an imbalance in oxidant/antioxidant markers, disruption of endogenous antioxidant defense, and an increase in oxidative factors (3). Among the highly oxidative products of oxidative stress are MDA and TOS. Instead, the depletion of antioxidant markers, such as TAC, GSH, catalase, and SOD, occurs under oxidative stress conditions (3). In addition, the PAB ratio has been investigated as an indicator of oxidative stress (53). Although most preclinical studies have shown the antioxidant effects of saffron and crocin, clinical trials are essential to determine their effectiveness in human studies.

The current updated meta-analysis of RCTs showed that intervention with saffron had beneficial effects on MDA, TOS, GPx, and TAC levels. Subgroup analysis revealed that a supplement dosage of >30 significantly reduced MDA levels in the trials. Interestingly, regarding TAC levels, it was found that in the trials prescribed, a dosage of <30 supplements was beneficial. In addition, contradictory results were observed regarding the effect of saffron supplementation duration on MDA and TAC levels in the subgroup analysis. Decreased levels of MDA were significantly evident in the administration duration of saffron <12 weeks, whereas increased levels of TAC were significant in the intervention for more than 12 weeks. Perhaps the reason for these contradictory results is the ambiguity of the findings due to the small sample size included in the subgroup analysis.

On the other hand, subgroup analysis of the results showed that the effect of age on oxidative stress markers was similar; therefore, under 50 years old, decreased MDA and increased TAC levels were significant compared to those above 50 years old. Severe oxidant/antioxidant imbalance in old age may have been an essential factor in the effects of saffron in seniors over 50 old. The results of the subgroup analysis also showed that crocin administration significantly reduced MDA and increased TAC levels compared with saffron administration. The results of the current meta-analysis are consistent with preclinical studies of the effectiveness of active saffron components such as crocin, crocetin, and safranal (63). Beneficial effects of crocin have been reported in various disorders such as asthma, COPD, PCOS, gastritis, and hepatitis (16, 17, 64). By upregulating the expression of mitochondrial antioxidant genes, crocin reduced ROS formation, decreased lipid peroxidation levels and MDA levels, increased TAC, and modified TOS (65).

The results of trial studies evaluating serum levels of TOS also showed the protective effects of crocin on serum levels. Two trials included in this study revealed that intervention with crocin reduced the serum TOS levels. No significant effect of saffron on the amount of PAB was observed. In fact, in two trial studies that examined the effect of saffron on PAB levels, it was not significant despite the decrease in serum PAB levels. The probable reason for the lack of significance in the results was the small sample size used in the current meta-analysis.

Various mechanisms have been proposed to explain the beneficial effects of saffron and its biologically active components in improving oxidative stress. In inflammatory diseases, ROS formation increases the production of intracellular advanced glycation end products, and activation of protein kinase C pathways occurs, leading to the activation of inflammatory signals, such as the NF-κB pathway, p38 mitogen-activated protein kinase (p38MAPK), Jun N-terminal kinases (JNK), and ER stress. The activation of inflammatory signaling cascades increases the synthesis and secretion of inflammatory cytokines, growth factors, eicosanoids, and chemokines. The antioxidant and anti-inflammatory effects of saffron are probably mediated by modulation of the following signaling pathways: NF-κB p65, protein kinase C (PKC), mitogen-activated protein kinases (MAPK/ERK), signal transducer and transcription activator 6 (STAT6), inducible nitric oxide synthase (iNOS), Ca2 + /calmodulin-dependent protein kinase 4 (CAMK4), ER stress markers, phosphoinositide-3-kinase (PI3K)/Akt, Nrf2, c-JNK, and high-mobility group box 1 (HMGB-1) pathways (15, 17, 59).

Although the current meta-analysis revealed the beneficial effects of saffron and crocin in clinical trial studies on oxidative stress markers such as TAC, MDA, and TOS, some limitations must be considered. First, the number of studies included in the current study, although higher than the previous study, was small for a more detailed analysis. Second, all the studies included in the meta-analysis were from Iran and could not be generalized to other nationalities. Third, some potential confounders that may have influenced the study results were not considered in the analysis of the trial studies, such as smoking, physical activity, and diet. Fourth, the nature of the diseases included in the trial study was different, which may have affected the study results. Finally, the lack of protocol registration was another limitation of the study.

In summary, the results of the current study showed that saffron and its active ingredients were able to establish a balance of oxidants/antioxidants in various disease conditions in trial studies. However, additional trial studies are necessary to reveal the effectiveness of saffron and its active ingredients on inflammatory and oxidative stress markers.

## Data availability statement

The original contributions presented in this study are included in the article/Supplementary material, further inquiries can be directed to the corresponding author.

## Ethics statement

This study was conducted after approved by the Ethics Committee of the Ardabil University of Medical Sciences (IR.ARUMS.MEDICINE.REC.1401.078).

## Author contributions

MA: conceptualization, methodology, data analysis, manuscript preparation, and revising the manuscript. AA and SI: data extraction and writing—original draft preparation. HG, AS, and MF: writing—original draft preparation, reviewing, and editing. All authors contributed to the article and approved the submitted version.
