# Supplementary figures and images for: Effect of saffron supplementation on oxidative stress markers (MDA, TAC, TOS, GPx, SOD, and pro-oxidant/antioxidant balance): An updated systematic review and meta-analysis of randomized placebo-controlled trials
Source: Front Med (Lausanne). 2023 Feb 1;10:1071514. doi: 10.3389/fmed.2023.1071514 (PMC9928952; doi:10.3389/fmed.2023.1071514)

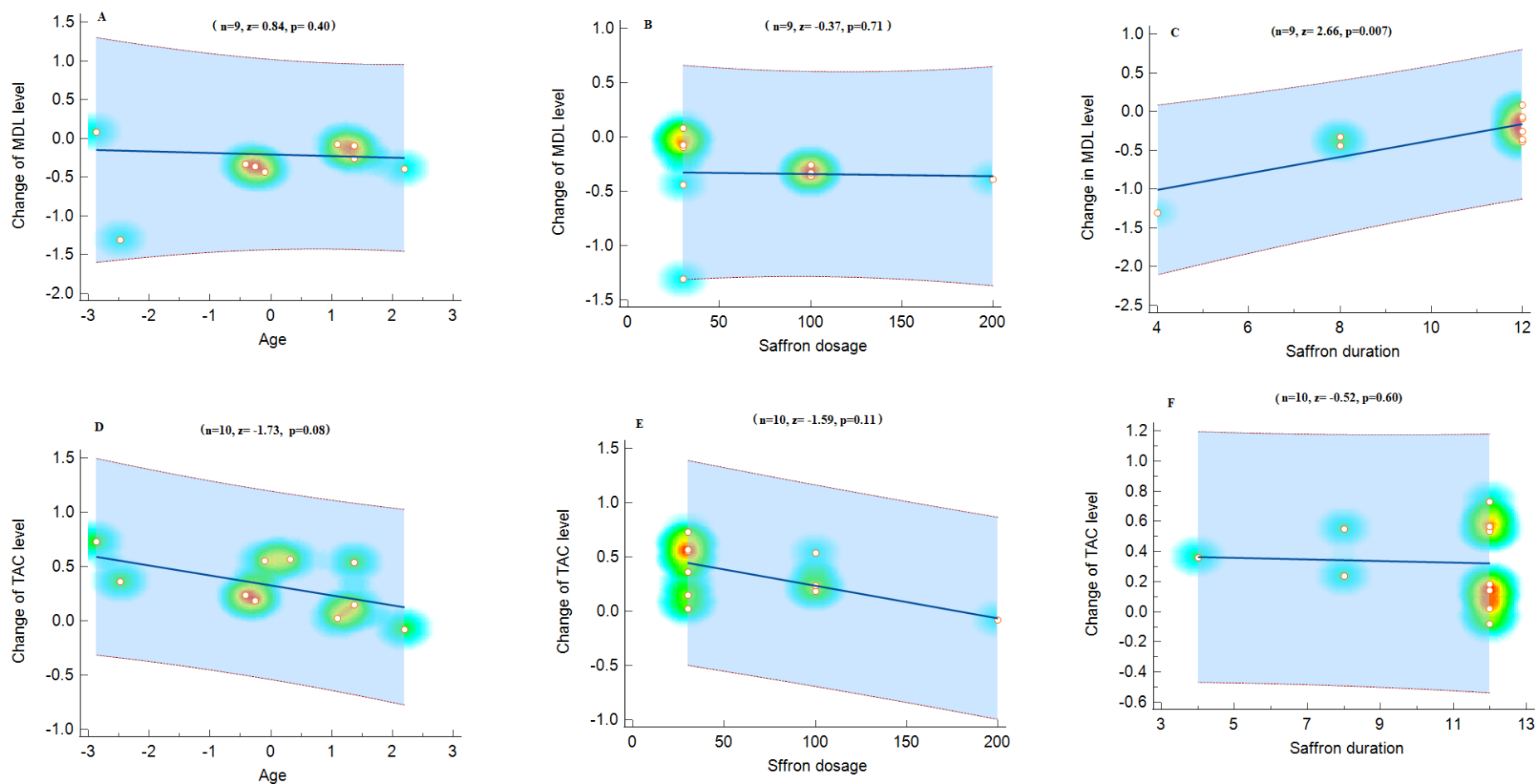

Supplementary Fig. 1. Meta-Regression scatter plots

Supplement: Supplementary file 1 [file Image_1.pdf]
