# Supplementary material for: Effect of saffron supplementation on oxidative stress markers (MDA, TAC, TOS, GPx, SOD, and pro-oxidant/antioxidant balance): An updated systematic review and meta-analysis of randomized placebo-controlled trials
Source: Front Med (Lausanne). 2023 Feb 1;10:1071514. doi: 10.3389/fmed.2023.1071514 (PMC9928952; doi:10.3389/fmed.2023.1071514)
Supplement: Supplementary file 2 [file Data_Sheet_1.pdf]

APPENDIX

The search term for Scopus was:

TITLE-ABS-KEY(Saffron OR crocin OR “crocus sativus” OR “saffron crocus” OR (crocus AND saffron))

TITLE-ABS-KEY("Oxidative Stress" OR antioxidant OR "antioxidant agent" OR "antioxidant nutrient" OR antioxidants OR "antioxidation agent" OR "antioxidation product" OR antioxidative OR antioxidant OR "phenolic antioxidant" OR superoxide OR glutathione OR "reactive oxygen species" OR Catalase OR "Oxygen Radical Absorbance" OR malondialdehyde OR "total antioxidant" OR "total oxidant" OR "lipid peroxide" OR "Nitric oxide")

TITLE-ABS-KEY(human OR clinical OR "placebo-controlled trial" OR "double-blind" OR "clinical trial" OR "Randomized Controlled Trial")

#1 AND #2 AND #3

The search term for Web of Science was:

TS=(Saffron OR (Crocus sativus) OR Safranal OR Crocin OR Crocetin OR Picrocrocin)

TS=((Oxidative Stress) OR antioxidant OR (antioxidant agent) OR (antioxidant nutrient) OR antioxidants OR (antioxidation agent) OR (antioxidation product) OR antioxidative OR antioxidant OR (phenolic antioxidant) OR superoxide OR glutathione OR (reactive oxygen species) OR Catalase OR (Oxygen Radical Absorbance) OR malondialdehyde OR (total antioxidant) OR (total oxidant) OR (lipid peroxide) OR (Nitric oxide))

TS=(human OR clinical OR (placebo-controlled trial) OR double-blind OR clinical trial OR (Randomized Controlled Trial))

#1 AND #2 AND #3

The search term for Pubmed was:

((Saffron OR crocin OR "crocus sativus" OR "saffron crocus" OR (crocus AND saffron))

(Oxidative Stress OR antioxidant OR antioxidant agent OR antioxidant nutrient OR antioxidants OR antioxidation agent OR antioxidation product OR antioxidative OR antioxidant OR phenolic antioxidant OR superoxide OR glutathione OR reactive oxygen species OR Catalase OR Oxygen Radical Absorbance OR malondialdehyde OR total antioxidant OR total oxidant OR lipid peroxide OR Nitric oxide)

(human OR clinical OR (placebo-controlled trial) OR double-blind OR clinical trial OR (Randomized Controlled Trial))

#1 AND #2 AND #3
